# Dissection of *PIK3CA* Aberration for Cervical Adenocarcinoma Outcomes

**DOI:** 10.3390/cancers13133218

**Published:** 2021-06-28

**Authors:** Tony K. H. Chung, Graeme Doran, Tak-Hong Cheung, So-Fan Yim, Mei-Yung Yu, Michael J. Worley, Kevin M. Elias, Aaron R. Thorner, Chandra Sekhar Pedamallu, Akinyemi I. Ojesina, Kei-Man Lau, Matthew D. Ducar, Raymond R. Y. Wong, Vivian W. Wang, Anwesha Nag, Bruce M. Wollison, Audrey Dalgarno, Jacqueline H. S. Lee, Suet-Ying Yeung, Lo Wong, Neil S. Horowitz, Michelle R. Davis, Shuk-On A. Leung, Yi Mu, Samuel C. Mok, Paul K. S. Chan, Michael S. Lawrence, Christopher P. Crum, Rossa W. K. Chiu, Ross S. Berkowitz, Yick-Fu Wong

**Affiliations:** 1 The Chinese University of Hong Kong, Prince of Wales Hospital, Shatin, Hong Kong; thcheung@cuhk.edu.hk (T.-H.C.); sfyim@cuhk.edu.hk (S.-F.Y.); myyu@cuhk.edu.hk (M.-Y.Y.); laukeiman@gmail.com (K.-M.L.); rrywong@uchicago.edu (R.R.Y.W.); jaclee@cuhk.edu.hk (J.H.S.L.); carolyeung@cuhk.edu.hk (S.-Y.Y.); lwong@cuhk.edu.hk (L.W.); paulkschan@cuhk.edu.hk (P.K.S.C.); rossachiu@cuhk.edu.hk (R.W.K.C.); 2 Firefly Bioworks, Inc., Cambridge, MA 02139, USA; doran.graeme@gmail.com; 3 Harvard Medical School, Brigham and Women’s Hospital, Boston, MA 02115, USA; mjworley@partners.org (M.J.W.J.); kelias@bwh.harvard.edu (K.M.E.); nhorowitz@mgh.harvard.edu (N.S.H.); mdavis31@bwh.harvard.edu (M.R.D.); soleung@bwh.harvard.edu (S.-O.A.L.); nhymu@channing.harvard.edu (Y.M.); ccrum@bwh.harvard.edu (C.P.C.); 4 Harvard Medical School, Dana-Farber Cancer Institute, Boston, MA 02115, USA; aaron_thorner@dfci.harvard.edu (A.R.T.); pcsmurali@gmail.com (C.S.P.); matt@ducar.com (M.D.D.); anwesha_nag@dfci.harvard.edu (A.N.); wollisonb@gmail.com (B.M.W.); audrey.dalgamo@gmail.com (A.D.); 5 Broad Institute of MIT and Harvard, Cambridge, MA 02142, USA; mslawrence@mgh.harvard.edu; 6 Department of Epidemiology, University of Alabama at Birmingham, Birmingham, AL 35294, USA; ojesina@uab.edu; 7 Mayo Clinic, Rochester, MN 55902, USA; vwwang@mayo.org; 8 MD Anderson Cancer Center, The University of Texas, Houston, TX 77030, USA; scmok@mdanderson.org; 9 Harvard Medical School, Massachusetts General Hospital, Boston, MA 02114, USA

**Keywords:** cervical adenocarcinoma, mutation, *PIK3CA*

## Abstract

**Simple Summary:**

There is limited information about genomic markers, especially for cervical adenocarcinoma treatment decisions. In this prospective study, it was found that nonsynonymous *PIK3CA* mutation detected in the patient’s circulating DNA collected before treatment or during follow-up was significantly associated with decreased progression-free survival or overall survival. It is the first indication of the predictive power of *PIK3CA* aberration in cervical adenocarcinoma. The work contributes to the development of liquid biopsies for the prolonged strategy of surveillance and indicates the possibility of tailoring management of this particular women’s cancer.

**Abstract:**

Personalized treatment of genetically stratified subgroups has the potential to improve outcomes in many malignant tumors. This study distills clinically meaningful prognostic/predictive genomic marker for cervical adenocarcinoma using signature genomic aberrations and single-point nonsynonymous mutation-specific droplet digital PCR (ddPCR). Mutations in *PIK3CA* E542K, E545K, or H1047R were detected in 41.7% of tumors. *PIK3CA* mutation detected in the patient’s circulating DNA collected before treatment or during follow-up was significantly associated with decreased progression-free survival or overall survival. *PIK3CA* mutation in the circulating DNA during follow-up after treatment predicted recurrence with 100% sensitivity and 64.29% specificity. It is the first indication of the predictive power of *PIK3CA* mutations in cervical adenocarcinoma. The work contributes to the development of liquid biopsies for follow up surveillance and a possibility of tailoring management of this particular women’s cancer.

## 1. Introduction

Cervical cancer remains the fourth most common cancer in women worldwide [1]. The two major types of cervical cancer are squamous cell carcinoma and adenocarcinoma, which are morphologically distinct. While most of the decline in cervical cancer can be attributed to a reduction in cervical squamous cell carcinoma, the incidence of cervical adenocarcinoma, absolute and relative to cervical squamous cell carcinoma, has been rising dramatically over the past few decades, in particular in young women [2]. The prognosis for advanced cervical adenocarcinoma is especially poor. Cervical cancer is almost always associated with infection of oncogenic types of human papillomavirus (HPV); however, HPV infection alone is insufficient for malignant transformation. Other genetic events independent or in conjunction with HPV infection are required [3]. There is great value in the characterization of the genomic signatures of cervical adenocarcinoma for the development of new prognostic/predictive markers and targeted therapeutic regimes to improve the outcome of women suffering from this disease.

This study addresses combining the signature genomic aberrations and patient clinical course to explore whether DNA markers can predict outcomes for this specific subtype of cervical cancer using a droplet digital polymerase chain reaction (ddPCR) platform.

## 2. Materials and Methods

### 2.1. Study Population

Twenty-four patients with primary cervical adenocarcinoma managed in the Department of Obstetrics and Gynaecology, The Chinese University of Hong Kong, Prince of Wales Hospital were included this study (Table 1). Surgery was offered to patients with early-stage disease unless there was a contraindication. Chemo-irradiation or primary radiotherapy was offered to patients with late-stage disease, or to those with a contraindication to radical surgery. Pelvic lymphadenectomy was performed as part of the surgical treatment procedure. The research protocol was approved by the Research Ethics Committee of The Chinese University of Hong Kong. Patients were provided with written informed consent for the use of their biological material for research purposes.

### 2.2. Sample Collection

Prior to any radiation or chemotherapy, surgical or biopsy tumor tissue specimens were embedded in OCT compound, snap-frozen in liquid nitrogen within one hour after collection and stored at −80 °C. Cryo-sections of tumor specimens were manually or laser-capture micro-dissected to achieve a tumor cell purity of 90% or greater. Patient blood samples were collected from patients prior to treatment, and at 6, 12, and 18 months after treatment.

Genomic DNA from tumor cells and blood cell pellet as self-control was isolated using AllPrep kit (Qiagen, Germantown, MD, USA), and quantified using Agilent 2100 Bioanalyzer (Agilent, Santa Clara, CA, USA). Plasma cell-free DNA (cfDNA) was isolated from plasma using QIAmp Circulating Nucleic Acid Kit (Qiagen, Germantown, MD). The range of DNA amount isolated from 3 mL of plasma was 18.3 to 702.5 (median 31.3) ng.

### 2.3. Digital Droplet Polymerase Chain Reaction (ddPCR)

A total of five single nucleotide variation (SNV) mutation sites from three genes were selected for ddPCR testing based on the cervical adenocarcinoma genomic landscape determined in previous studies, the yield of cfDNA, as well as the availability of established ddPCR mutation assays [4,5,6,7,8,9]. The ddPCR reactions contained 10 μL of 2×ddPCR supermix, 1 μL of 20× primer/probe mix, and 9 μL of diluted DNA (3 ng). The 20 μL reaction mix was transferred to a cartridge for a QX200 droplet generator (Bio-Rad, Hercules, CA, USA) followed by 70 μL of droplet generation oil into oil wells. After droplet generation, 40 μL of the reaction were then transferred to a 96-well plate and the plate was heat-sealed with sealing foil sheets. The optimized cycling conditions were 95 °C for 10 min, 40 cycles of 94 °C for 30 s, annealing temperature for 1 min, followed by 98 °C for 10 min and a hold at 4 °C using thermal cycler C1000 (Bio-Rad, Hercules, CA, USA). The annealing/extension temperature of *PIK3CA* E542K, *PIK3CA* E545K, *PIK3CA* H1047R, *KRAS* G12V and *KRT6A* F249L were 58.7 °C, 61 °C, 61 °C, 58.7 °C, and 57.9 °C, respectively. After PCR amplification, the plate was read by the QX200 droplet reader (Bio-Rad, Hercules, CA, USA) for analysis. The DNA targets were quantified using QuantaSoft Software (Bio-Rad, Hercules, CA, USA). The results are reported as copies of mutant allele per μL of reaction. To verify the ddPCR specificity, 10 g blocks gene fragments containing the mutant or wildtype sequence for the five targeted mutation sites were utilized as templates (Integrated DNA Technologies Inc., Coralville, IA, USA) (Supplementary Table S1), and 20,000 copies of the gene fragments were used for each assay.

### 2.4. Clinicopathological Data Collection and Statistical Analyses

Clinicopathological data from each patient were collected from medical records for statistical analysis. The associations between gene mutations and the clinicopathological features were analyzed by a Kaplan–Meier survival curve and log-rank test, Cox proportional hazards regression analysis, Fisher’s exact test, Binary logistic regression analysis, and receiver operating characteristic (ROC) curve analysis using MedCalc (Version 19.5.1) software. The results derived from the Kaplan–Meier survival curve and log-rank test in part was also verified using SPSS (Version 25) and SAS (University Edition) software. A two-sided *p* value < 0.05 was considered statistically significant.

## 3. Results

Using single-point mutation-specific ddPCR, a total of five nonsynonymous single nucleotide substitution mutation spots were measured in tumor DNA and circulating DNA (Supplementary Table S2). At least one of three *PIK3CA* missense mutation spots was detected in 10 (41.7%) of 24 tumor DNA samples, 7 (30.4%) of 23 circulating DNA samples collected before treatment, and 12 (29.3%) of 41 DNA samples collected during follow-up after treatment. There was no statistically significant difference among the three positions of the *PIK3CA* mutation detected in tumor DNA and in circulating DNA collected before treatment or during follow-up (*p* > 0.05). *KRT6A* F249L mutation was detected in 12 (50%) tumor DNA samples, seven (30.4%) circulating DNA samples collected before treatment, and 11(29.7%) circulating DNA samples collected during follow-up. *KRAS* G12V mutation was detected in four (25%) tumor DNA samples and not detected in circulating DNA samples collected before treatment or during follow-up.

**Figure 1:**
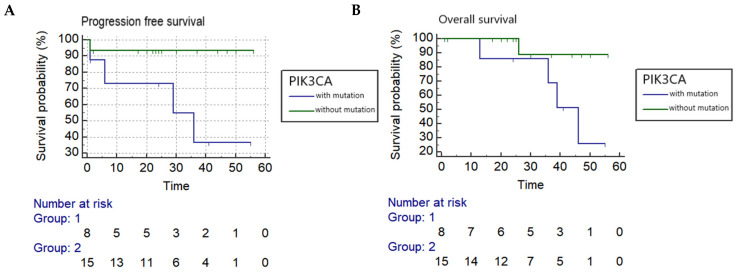
Kaplan–Meier analysis (**A**) Progression-free survival correlated to *PIK3CA* in circulating DNA collected before treatment in cervical adenocarcinoma (*p* = 0.0291) (hazard ratio = 7.7068). (**B**) Overall survival correlated to *PIK3CA* in circulating DNA collected before treatment in cervical adenocarcinoma (*p* = 0.0499) (hazard ratio = 6.6105).

Kaplan–Meier log-rank analyses found a *PIK3CA* mutation in circulating DNA collected before treatment was associated with a statistically significant worse progression-free and overall survival with a hazard ratio of 7.7 and 6.6, respectively (PFS: *p* = 0.0291 and OS: *p* = 0.0499) (Figure 1), while reduced OS was associated with *PIK3CA* mutation in circulating DNA collected during follow-up after treatment (*p* = 0.0429). Tumor recurrence was also significantly correlated to both PFS and OS (*p* = 0.0001 and *p* = 0.0026, respectively). In addition, OS was significantly associated with pelvic LN metastasis (*p* = 0.0143), and reduced PFS and OS were significantly correlated to a late stage of cancer (*p* = 0.0036 and *p* = 0.0007, respectively) (Table 2).

In Cox proportional hazards regression analysis, the factors significantly associated with survival included the presence of *PIK3CA* mutation in circulating DNA collected before treatment, stage, tumor size and recurrence (*p* = 0.015, *p* = 0.0034, *p* = 0.016 and *p* < 0.0001, respectively) (Supplementary Table S3). The factors significantly correlated to recurrence included *PIK3CA* mutation in circulating DNA collected during follow-up and tumor size (*p* = 0.0114 and *p* = 0.0185, respectively) while late stage was marginally associated with recurrence (*p* = 0.0561) (Supplementary Table S4).

Correlation analysis using Fisher’s exact test showed that patient survival was significantly associated with stage, recurrence, and *PIK3CA* mutation status in circulating DNA collected before treatment (*p* = 0.0145, *p* = 0.005, and *p* = 0.0329, respectively), while recurrence was significantly associated with survival and *PIK3CA* mutation status in circulating DNA collected during follow-up (*p* = 0.005 and *p* = 0.0325, respectively) (Supplementary Table S5).

Binary logistic regression analysis showed that the stage (*p* = 0.018), tumor size (*p* = 0.039), and *PIK3CA* mutation in circulating DNA collected before treatment (*p* = 0.035), were significantly associated with survival.

**Figure 2:**
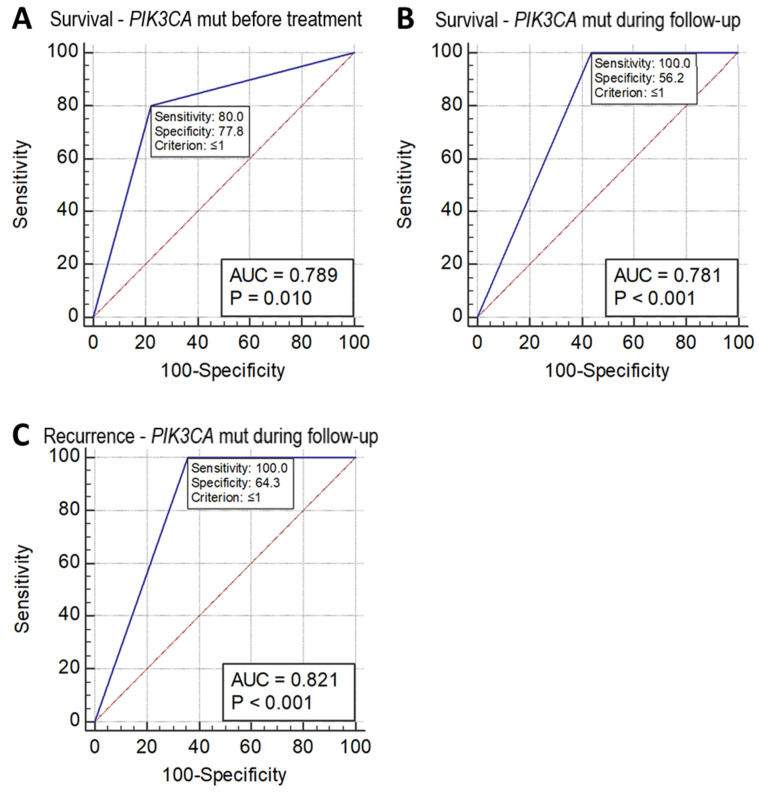
ROC chart. (**A**) Survival of cervical adenocarcinoma predicted by *PIK3CA* mutation status as detected in circulating DNA collected before treatment. (**B**) Survival of cervical adenocarcinoma predicted by *PIK3CA* mutation status as detected in circulating DNA collected during follow-up after treatment. (**C**) Recurrence of cervical adenocarcinoma predicted by *PIK3CA* mutation status as detected in circulating DNA collected during follow-up after treatment.

Receiver operating characteristic (ROC) analysis of sensitivity and specificity of these missense mutation measurements in the prediction of patient survival and recurrence showed that the mutation status of *PIK3CA* detected in circulating DNA collected before treatment and during follow-up after treatment could predict a reduction in survival with a sensitivity and specificity of 80.0% and 77.8% (*p* = 0.010, AUC = 0.789), and 100.0% and 56.2% (*p* = 0.0001, AUC 0.781), respectively. *PIK3CA* mutations detected in circulating DNA collected during follow-up after treatment could predict recurrence with a sensitivity and specificity of 100% and 64.3%, respectively (*p* = 0.001, AUC = 0.821) (Figure 2). However, the ROC analysis did not show *p* < 0.05 in the prediction of either the recurrence or survival status in the mutation of *PIK3CA* in tumor tissue DNA alone or of the other two SNVs (*KRT6A* F249L and *KRAS* G12V) in either tumor tissue DNA, or circulating DNA collected before treatment or during follow-up.

## 4. Discussion

In a prior retrospective study, we identified mutations in *FAT1*, *ARID1A*, *ERBB2*, and PIK3CA in whole-exome sequencing of 15 patients with cervical adenocarcinoma [8]. We then assessed a second cohort of 24 patients from Hong Kong as a prospective validation set and verified recurrent genomic aberrations in this type of cervical cancer [9].

Due to the limited quantity of circulating DNA preparations, we performed the ddPCR analysis to five missense single nucleotide substitution mutation spots of three genes in the present study. The most significant finding in the study was that the measurement of three single-point nonsynonymous mutations in *PIK3CA*: E542K, E545K, and H1047R using ddPCR is predictive of survival in a subgroup of cervical adenocarcinoma. We noted that in six of seven (85.7%) cases with recurrence, there was a *PIK3CA* mutation detected in tumor tissue DNA or circulating cfDNA collected before the recurrence occurred clinically (Supplementary Table S6). One case had a recurrence without a *PIK3CA* mutation detected in her tumor DNA or circulating cfDNA collected before treatment; however, this case did not have cfDNA samples collected during follow-up for testing the *PIK3CA* mutation. Therefore, we could not exclude the possibility that a *PIK3CA* mutation had existed before recurrence occurred clinically. Although the numbers are small, the high frequency of *PIK3CA* variants identified in samples before recurrence is noteworthy. ddPCR is increasingly available in clinical care. Further study is warranted to validate our findings; however, ddPCR detection of PIK3CA could be implemented in clinical practice for the early identification of recurrent disease, which may have prognostic significance.

Over 80% of somatic missense mutations in *PIK3CA* are found in the kinase and helical domains of the *PIK3CA* subunit, and cluster in three “hotspots”: E542K, E545K (in kinase domain), and H1047R (in helical domain) [10]. Mutational activation of *PIK3CA* detected in tumor tissue has been found in association with both an adverse clinical outcome of patients in multiple solid tumors and can predict a favorable response to *PIK3CA* inhibitors [10]. However, there is disagreement as to whether the *PIK3CA* mutation status correlates with patient survival in cervical cancer in previous reports [11,12,13,14,15,16,17,18,19] (Supplemental Table S7). The discrepancy of the PIK3CA mutation detection rate in tumor tissue of cervical adenocarcinoma among these reports were attributed to the differing purity of tumor cells in a sample, differing sensitivity of the measurement, or differing populations and regions as well as differing sample sizes. It is desirable to conduct a collaborative study in multiple centers in different regions with different races using a standardized mutation detection method in the future. These studies only tested *PIK3CA* mutations in tumor DNA but not circulating DNA and did not clearly analyze different sub-types of cervical cancer separately, while our work contributes novel data to this assessment. The correlation between the findings of mutations detected in cfDNA in other malignant tumors and negative prognosis was also reported. In patients of advanced non-small-cell lung cancer (NSCLC) with tissue epidermal growth factor receptor (*EGFR*) T790M-positive, an absence of detectable plasma T790M at baseline is associated with longer progression-free survival, which may be attributed to a lower disease burden [20]. In patients with estrogen receptor-positive advanced metastatic breast cancer (ER+ mBC) in the BEECH study, it was shown that early on-treatment ctDNA dynamics are a surrogate for progression-free survival. Dynamic ctDNA assessment has the potential to substantially enhance early drug development [21].

In the present study, somatic point mutations in *KRAS* appeared in a low frequency in both tumor and circulating DNA samples. *KRAS* is a dominant oncogene with alterations found in both COSMIC and cBioPortal. There are multiple hotspot mutations in *KRAS*, but only one spot, p.G12V, was examined in the present study due to the limited amount of circulating DNA available in the sample pool. A *KRT6A* point substitution mutation was detected in 12 of 24 (50%) patients in this study. Keratin 6A is a type II cytokeratin hyperexpressed keratin 6A in lung adenocarcinoma that promotes lung cancer proliferation and metastasis via epithelial–mesenchymal transition and cancer stem cells transformation [22]. Although the mutation was not associated with clinicopathological features, it may be worthwhile to explore its pathologic functions in cervical adenocarcinoma and the potential to be a therapeutic target in this disease.

One of the limitations of the current work is the small sample size. While five major medical centers were included in this study, the final results were only able to be obtained from Prince of Wales Hospital (PWH), Hong Kong, resulting in a smaller case series.

However, despite the small sample size, the results were significant and thus warrant further investigation. Some questions remain: (1) are the associations valid across multiple cancer centers in different regions and different demographics, (2) can ddPCR of the *PIK3CA* mutation be implemented for accurate and affordable disease monitoring, and (3) can an appropriate *PIK3CA* inhibitor improve outcomes for a late stage of cancer with *PIK3CA* mutation detectable? Hopefully, a subgroup of particularly advanced cervical adenocarcinoma patients can benefit from this specialized prognosis, treatment and management. When designing continuing prospective validation studies, a priori power analysis and sample calculation are suggested to achieve the most reliable output.

## 5. Conclusions

This translational study identified that the detection of *PIK3CA* mutations in three hotspots and combining tumor and circulating DNA assessment using robust ddPCR was predictive of a reduction in both progression-free and overall survival in a clinically important sub-group of cervical adenocarcinoma. The stratification of patients with cervical adenocarcinoma, regardless of stage, based on the *PIK3CA* mutation could be implemented in a clinical setting able to perform ddPCR assays on liquid biopsies, and may offer the possibility of tailoring management.

## Figures and Tables

**Table 1:** Clinicopathological features of cervical adenocarcinoma.

Case	Code	Age	Stage	Grade	Size	LN	LVSI	Recur	Status	S/F Time	p16	HPV Genotype
1	C1062	47	IB1	2	2	(−)	n/a	(−)	Alive	55	n/a	Undetected
2	C1071	35	IB2	3	4	n/a	(+)	(−)	Alive	58	(−)	Undetected
3	C1072	49	IB2	1	1	n/a	(−)	n/a	Alive	2	(−)	HPV18
4	C1074	63	IB1	3	2	(−)	(−)	(−)	Alive	56	(−)	HPV18
5	C1080	35	IB1	2	3	(−)	(−)	(+)	Alive	47	n/a	HPV18
6	C1082	75	IIB	2	4	(+)	(+)	(+)	Died	39	(+)	HPV52
7	C1084	38	IB1	2	3	(−)	(−)	(+)	Died	46	n/a	HPV16
8	C1094	54	IB2	2	1	n/a	n/a	(−)	Alive	50	(+)	HPV18
9	C1106	53	IV	2	5	n/a	n/a	(+)	Died	36	n/a	HPV18
10	C1108	43	IB1	2	1	(−)	(−)	(−)	Alive	47	(+)	HPV16
11	C1112	57	IB1	3	2	(−)	(−)	(+)	Alive	44	(+)	HPV18
12	C1119	43	IIB	2	4	n/a	n/a	(−)	Alive	41	(+)	HPV18
13	C1126	71	IIB	2	4	n/a	n/a	(+)	Died	13	n/a	Undetected
14	C1128	34	IIB	2	1	n/a	(−)	n/a	Alive	1	(+)	HPV18
15	C1129	42	IIB	2	2	n/a	(−)	(−)	Alive	30	(+)	HPV18
16	C1135	40	IV	2	1	n/a	(+)	(+)	Died	26	n/a	Undetected
17	C1136	46	IB1	2	1	(−)	(−)	(−)	Alive	37	(+)	HPV16
18	C1138	49	IB1	2	1	(−)	(−)	(−)	Alive	37	(+)	HPV16
19	C1165	55	IB1	3	1	(−)	(−)	(−)	Alive	25	n/a	HPV18
20	C1169	48	IB2	2	1	(−)	(−)	(−)	Alive	24	(+)	HPV16
21	C1172	57	IB1	2	2	(−)	(−)	(−)	Alive	24	(+)	Undetected
22	C1177	57	IB1	2	3	(−)	(−)	(−)	Alive	17	n/a	HPV18
23	C1181	37	IB1	2	1	n/a	n/a	(−)	Alive	22	(+)	HPV45
24	C1187	51	IB2	2	3	(+)	(+)	(−)	Alive	20	n/a	HPV16

NOTE: Stage, clinical staging (FIGO); Grade, pathological grading (WHO); Size, largest tumor diameter (cm); Recur, tumor recurrence; Status, clinical status, alive or dead; S/F time, survival time of deceased patients/follow-up time of alive patients (months); p16: p16 immunochemistry staining in tumor section; HPV genotype, HPV infection and type tested using linear array HPV genotyping test in tumor DNA; HPV in WES, HPV DNA sequences checked in tumor’s WES profile; (+), positive; (−), negative; n/a: no information available. Abbreviations: LN, pelvic lymph node metastasis; LVSI, lymph vascular space invasion. NOTE: Stage, clinical staging (FIGO); Grade, pathological grading (WHO); Size, largest tumor diameter (cm); Recur, tumor recurrence; Status, clinical status, alive or dead; p16: p16 immunochemistry staining in tumor section; HPV genotype, HPV infection and type tested using linear array HPV genotyping test in tumor DNA; HPV in WES, HPV DNA sequences checked in tumor’s WES profile; (+), positive; (−), negative; n/a: no information available. Abbreviations: LN, pelvic lymph node metastasis; LVSI, lymph vascular space invasion.

**Table 2:** Survival analysis related to clinicopathological features and gene mutations in cervical adenocarcinoma using Kaplan–Meier survival curve and log-rank test.

Features and SNV Mutations	PFS		OS	
	*p* Value	Hazard Ratio	*p* Value	Hazard Ratio
Age (Grouped) (Year) 1 ≤ 50 2 > 50	0.2681	0.3860	0.2872	0.3943
Stage (Grouped) FIGO staging 1 = stage IA-IIA 2 = stage IIB-IV	**0.0036**	12.2162	**0.0007**	15.2896
Grade (Grouped) WHO grading 1 = grade1,2 2 = grade3	0.5356	n/a	n/a	n/a
Tumor size (cm)	0.0784	n/a	0.2051	n/a
Pelvic LN metastasis	0.1284	6.4667	0.0143	13.0000
LVSI	0.1838	4.3955	0.2448	3.7600
Recurrence	**0.0001**	n/a	**0.0026**	n/a
P16	0.5637	n/a	0.5271	n/a
HPV	0.2156	0.3443	0.3057	0.4069
*PIK3CA*—T	0.6505	1.5000	0.8171	1.2340
*PIK3CA*—B	**0.0291**	7.7068	**0.0499**	6.6105
*PIK3CA*—F	0.0679	n/a	**0.0429**	n/a
*KRAS*—T	0.4792	2.1376	0.4792	2.1376
*KRAS*—B	n/a	n/a	n/a	n/a
*KRAS*—F	0.5202	n/a	0.4183	n/a
*KRT6A*—T	0.3978	0.4762	0.5151	0.5579
*KRT6A*—B	0.8829	0.8501	n/a	n/a
*KRT6A*—F	0.4448	0.4067	0.7033	0.6299

NOTE: SNV, single nucleotide variation; PFS, progression-free survival; OS, overall survival; LVSI, lymphovascular space invasion;—B, mutation detected in circulating DNA collected before treatment;—F, mutation detected in circulating DNA collected during follow-up after treatment;—T, mutation detected in tumor tissue DNA, Bold values represent *p* Values < 0.05

## Data Availability

Data is contained within the article or Supplementary Materials.

## Supplementary Materials

The following are available online at https://www.mdpi.com/article/10.3390/cancers13133218/s1. Table S1. DNA sequences of gBlock gene fragments used as controls for each mutation site. Table S2. Gene mutation copy (Copies/ng) detected in tumor tissue DNA, and circulating DNA collected before treatment and during the follow-up period. Table S3. Cox regression analysis of survival related to clinicopathological features and gene mutations shown as *p*-value in cervical adenocarcinoma. Table S4. Cox regression analysis of recurrence related to clinicopathological features and gene mutations shown as *p*-value in cervical adenocarcinoma. Table S5. Association of survival and with clinicopathological features and gene mutations in cervical adenocarcinoma analyzed using Fisher’s exact test shown as *p*-value. Table S6. *PIK3CA* mutation detection in cervical adenocarcinoma with recurrence. Table S7. *PIK3CA* mutation and clinical outcome in cervical adenocarcinoma in previous reports.
