# Supplementary material for: Dissection of PIK3CA Aberration for Cervical Adenocarcinoma Outcomes
Source: Cancers (Basel). 2021 Jun 28;13(13):3218. doi: 10.3390/cancers13133218 (PMC8269188; doi:10.3390/cancers13133218)
Supplement: Supplementary file 1 [file cancers-13-03218-s001.zip › cancers-1192581-supplementary.pdf]

cancers

MDPI

Supplementary

Dissection of *PIK3CA* Aberration for Cervical Adenocarcinoma Outcomes

Tony K. H. Chung et al.

Table S1. DNA sequences of gBlock gene fragments used as controls for each mutation site.

Mutation Spot	Sequence of Control Gene Fragment
PIK3CA p.E542K	CCCATGAGACATACAAAAAGGTAATGCCGCCTCGCTAGGTGAGCTACAGCTCGATTGT<br>CACGTTAAGCTGGCCAAAATGTATTTGCTTTTTCTGTAAATCATCTGTGAATCCAGAGG<br>GGAAAAATATGACAAAGAAAGCTATATAAGATATTATTTTATTTTACAGAGTAACAGACT<br>AGCTAGAGACAATGAATTAAGGGAAAATGACAAAGAACAGCTCAAAGCAATTTCTACA<br>CGAGATCCTCTCTCT**A**AAATCACTGAGCAGGAGAAAGATTTTCTATGGAGTCACAGGT<br>AAGTGCTAAAATGGAGATTCTCTGTTTCTTTTTCTTTATTACAGAAAAAAATAACTGAATT<br>TGGCTGATCTCAGCATGTTTTTACCATACCTATTGGAATAAATAAAGCAGAATTTACAT<br>GATTTTTAAACTATAAACATTGCCTTTTTAAAAACAATGGTTGTAAATTGATATTTGTGG<br>GTCTCGACTATACGCCCGTTTTCGGATC
WT for *PIK3CA* p.E542K	CCCATGAGACATACAAAAAGGTAATGCCGCCTCGCTAGGTGAGCTACAGCTCGATTGT<br>CACGTTAAGCTGGCCAAAATGTATTTGCTTTTTCTGTAAATCATCTGTGAATCCAGAGG<br>GGAAAAATATGACAAAGAAAGCTATATAAGATATTATTTTATTTTACAGAGTAACAGACT<br>AGCTAGAGACAATGAATTAAGGGAAAATGACAAAGAACAGCTCAAAGCAATTTCTACA<br>CGAGATCCTCTCTCT**G**AAATCACTGAGCAGGAGAAAGATTTTCTATGGAGTCACAGGT<br>AAGTGCTAAAATGGAGATTCTCTGTTTCTTTTTCTTTATTACAGAAAAAAATAACTGAATT<br>TGGCTGATCTCAGCATGTTTTTACCATACCTATTGGAATAAATAAAGCAGAATTTACAT<br>GATTTTTAAACTATAAACATTGCCTTTTTAAAAACAATGGTTGTAAATTGATATTTGTGG<br>GTCTCGACTATACGCCCGTTTTCGGATC
PIK3CA p.E545K	CCCATGAGACATACAAAAAGGTAATGCCGCCTCGCTAGGTGAGCTACAGCTCGATTGT<br>CACGTTAAGCTGGCCTTGCTTTTTCTGTAAATCATCTGTGAATCCAGAGGGGAAAAATA<br>TGACAAAGAAAGCTATATAAGATATTATTTTATTTTACAGAGTAACAGACTAGCTAGAGA<br>CAATGAATTAAGGGAAAATGACAAAGAACAGCTCAAAGCAATTTCTACACGAGATCCTC<br>TCTCTGAAATCACT**A**AGCAGGAGAAAGATTTTCTATGGAGTCACAGGTAAGTGCTAAAA<br>TGGAGATTCTCTGTTTCTTTTTCTTTATTACAGAAAAAAATAACTGAATTTGGCTGATCTC<br>AGCATGTTTTTACCATACCTATTGGAATAAATAAAGCAGAATTTACATGATTTTTAAACT<br>ATAAACATTGCCTTTTTAAAAACAATGGTTGTAAATTGATATTTGTGGAAAATCATGGTC<br>TCGACTATACGCCCGTTTTCGGATC
WT for *PIK3CA* p.E545K	CCCATGAGACATACAAAAAGGTAATGCCGCCTCGCTAGGTGAGCTACAGCTCGATTGT<br>CACGTTAAGCTGGCCTTGCTTTTTCTGTAAATCATCTGTGAATCCAGAGGGGAAAAATA<br>TGACAAAGAAAGCTATATAAGATATTATTTTATTTTACAGAGTAACAGACTAGCTAGAGA<br>CAATGAATTAAGGGAAAATGACAAAGAACAGCTCAAAGCAATTTCTACACGAGATCCTC<br>TCTCTGAAATCACT**G**AGCAGGAGAAAGATTTTCTATGGAGTCACAGGTAAGTGCTAAA<br>ATGGAGATTCTCTGTTTCTTTTTCTTTATTACAGAAAAAAATAACTGAATTTGGCTGATCT<br>CAGCATGTTTTTACCATACCTATTGGAATAAATAAAGCAGAATTTACATGATTTTTAAAC<br>TATAAACATTGCCTTTTTAAAAACAATGGTTGTAAATTGATATTTGTGGAAAATCATGGT<br>CTCGACTATACGCCCGTTTTCGGATC
PIK3CA p.H1047R	CCCATGAGACATACAAAAAGGTAATGCCGCCTCGCTAGGTGAGCTACAGCTCGATTGT<br>CACGTTAAGCTGGCCATCTAGCTATTCGACAGCATGCCAATCTCTTCATAAATCTTTTC<br>TCAATGATGCTTGGCTCTGGAATGCCAGAACTACAATCTTTTGATGACATTGCATACAT<br>TCGAAAGACCCTAGCCTTAGATAAAACTGAGCAAGAGGCTTTGGAGTATTTCATGAAA

	CAAATGAATGATGCAC**G**TCATGGTGGCTGGACAACAAAAATGGATTGGATCTTCCACA<br>CAATTAAACAGCATGCATTGAACTGAAAAGATAACTGAGAAAATGAAAGCTCACTCTGG<br>ATTCCACACTGCACTGTTAATAACTCTCAGCAGGCAAAGACCGATTGCATAGGAATTG<br>CACAATCCATGAACAGCATTAGAATTTACAGCAAGAACAGAAATAAAATACTATATAATT<br>TAGGTCTCGACTATACGCCCGTTTTCGGATC
**WT for *PIK3CA* p.H1047R**	CCCATGAGACATACAAAAAGGTAATGCCGCCTCGCTAGGTGAGCTACAGCTCGATTGT<br>CACGTTAAGCTGGCCATCTAGCTATTCGACAGCATGCCAATCTCTTCATAAATCTTTTC<br>TCAATGATGCTTGGCTCTGGAATGCCAGAACTACAATCTTTTGATGACATTGCATACAT<br>TCGAAAGACCCTAGCCTTAGATAAAACTGAGCAAGAGGCTTTGGAGTATTTCATGAAA<br>CAAATGAATGATGCAC**A**TCATGGTGGCTGGACAACAAAAATGGATTGGATCTTCCACA<br>CAATTAAACAGCATGCATTGAACTGAAAAGATAACTGAGAAAATGAAAGCTCACTCTGG<br>ATTCCACACTGCACTGTTAATAACTCTCAGCAGGCAAAGACCGATTGCATAGGAATTG<br>CACAATCCATGAACAGCATTAGAATTTACAGCAAGAACAGAAATAAAATACTATATAATT<br>TAGGTCTCGACTATACGCCCGTTTTCGGATC
***KRAS* p.G12V,**	CCCATGAGACATACAAAAAGGTAATGCCGCCTCGCTAGGTGAGCTACAGCTCGATTGT<br>CACGTTAAGCTGGCCTTATCTTGTAATAAGTACTCATGAAAATGGTCAGAGAAACCTTT<br>ATCTGTATCAAAGAATGGTCCTGCACCAGTAATATGCATATTAAAACAAGATTTACCTC<br>TATTGTTGGATCATATTCGTCCACAAAATGATTCTGAATTAGCTGTATCGTCAAGGCAC<br>TCTTGCCTACGCCA**A**CAGCTCCAACTACCACAAGTTTATATTCAGTCATTTTCAGCAGG<br>CCTTATAATAAAAATAATGAAAATGTGACTATATTAGAACATGTCACACATAAGGTTAAT<br>ACACTATCAAATACTCCACCAGTACCTTTTAATACAAACTCACCTTTATATGAAAAATTA<br>TTTCAAAATACCTTACAAAATTCAATCATGAAAATTCCAGTTGACTGCAGACGTGTAGG<br>TCTCGACTATACGCCCGTTTTCGGATC
**WT for *KRAS* p.G12V,**	CCCATGAGACATACAAAAAGGTAATGCCGCCTCGCTAGGTGAGCTACAGCTCGATTGT<br>CACGTTAAGCTGGCCTTATCTTGTAATAAGTACTCATGAAAATGGTCAGAGAAACCTTT<br>ATCTGTATCAAAGAATGGTCCTGCACCAGTAATATGCATATTAAAACAAGATTTACCTC<br>TATTGTTGGATCATATTCGTCCACAAAATGATTCTGAATTAGCTGTATCGTCAAGGCAC<br>TCTTGCCTACGCCA**C**CAGCTCCAACTACCACAAGTTTATATTCAGTCATTTTCAGCAGG<br>CCTTATAATAAAAATAATGAAAATGTGACTATATTAGAACATGTCACACATAAGGTTAAT<br>ACACTATCAAATACTCCACCAGTACCTTTTAATACAAACTCACCTTTATATGAAAAATTA<br>TTTCAAAATACCTTACAAAATTCAATCATGAAAATTCCAGTTGACTGCAGACGTGTAGG<br>TCTCGACTATACGCCCGTTTTCGGATC
***KRT6A* p.F249L**	CCCATGAGACATACAAAAAGGTAATGCCGCCTCGCTAGGTGAGCTACAGCTCGATTGT<br>CACGTTAAGCTGGCCGTTCTGGAAACAAAGTGGACCCTGCTGCAGGAGCAGGGCACC<br>AAGACTGTGAGGCAGAACCTGGAGCCGTTGTTCGAGCAGTACATCAACAACCTCAGG<br>AGGCAGCTGGACAGCATTGTCGGGGAACGGGGCCGCCTGGACTCAGAGCTCAGAGG<br>CATGCAGGACCTGGTGGAGGAC**C**TCAAGAACAAGTGAGTTAAACAGGAGAAATGGAC<br>TCAGTTTCCTGCAGCACACACTTCAAGACTATTGGGTGACCAGGGCCAGAAGGGGGT<br>AATATTCCTAACAACCCATGTCCGTGGAAATGAAGAGCACAAATTAGTCCCTAGGAGC<br>AAACCTGCAAGAACCACAGATGGTTATGGGAGGATGGGGAGATTAAAGAAGTGGCAA<br>ATTTAGCAGAAGCAGGTCTCGACTATACGCCCGTTTTCGGATC
**WT for *KRT6A* p.F249L**	CCCATGAGACATACAAAAAGGTAATGCCGCCTCGCTAGGTGAGCTACAGCTCGATTGT<br>CACGTTAAGCTGGCCGTTCTGGAAACAAAGTGGACCCTGCTGCAGGAGCAGGGCACC<br>AAGACTGTGAGGCAGAACCTGGAGCCGTTGTTCGAGCAGTACATCAACAACCTCAGG<br>AGGCAGCTGGACAGCATTGTCGGGGAACGGGGCCGCCTGGACTCAGAGCTCAGAGG<br>CATGCAGGACCTGGTGGAGGAC**T**TCAAGAACAAGTGAGTTAAACAGGAGAAATGGAC<br>TCAGTTTCCTGCAGCACACACTTCAAGACTATTGGGTGACCAGGGCCAGAAGGGGGT<br>AATATTCCTAACAACCCATGTCCGTGGAAATGAAGAGCACAAATTAGTCCCTAGGAGC<br>AAACCTGCAAGAACCACAGATGGTTATGGGAGGATGGGGAGATTAAAGAAGTGGCAA<br>ATTTAGCAGAAGCAGGTCTCGACTATACGCCCGTTTTCGGATC

Table S2. Gene mutation copy (Copies/ng) detected in tumor tissue DNA, and circulating DNA collected before treatment and during the follow-up period.

**Case**	***PIK3CA* E542K**					***PIK3CA* E545K**					***PIK3CA* H1047R**				
	**T**	**0 m**	**6 m**	**12 m**	**18 m**	**T**	**0 m**	**6 m**	**12 m**	**18 m**	**T**	**0 m**	**6 m**	**12 m**	**18 m**
1	0	0		0	0	0	0		0	0	0	0.67			
2	0			0	0	0.6			0	0	0.4			0	0
3	0	0				0	0				0	0			
4	0	0		0	0	0.47	0		0	0	0.93	0		0	0
5	0	0		0		1	0		0		0	0		0	
6	0	0	0	0	0	140	1.27	0	0.47	0.4	0.4	0.47	0.47	0.47	0
7	0	0	0	0		0	0	0.4	0.47		0.4	0.53	0	0	
8	0	0			0	0	0			0	0	0			0
9	0	0.53	0	0.53		0	0	0	0		0	0	0	0	
10	0	0			0	0	0			0	0	0			0
11	0	0	0	0.47		0.67	0	0	0		0	0	0.87	0.53	
12	0	0				0	0				0	0.47			
13	60.67	3				0	0				0	0.67			
14	0	5.33				0	0				0	0			
15	0	0	0	0	0	0	0	0	0	0	0.53	0	0	0	0
16	0	0				0	0				0	0			
17	0.53	0			0	0	0			0	0	0			2
18	0	0	0.67	0	0	0	0	0	0	0	0	0	0	0	0.67
19	0	0	0	0	0	0	0	0	0	0.53	0	0	0	0	0.67
20	0	0	0	0	0	0	0.6	0.67	0	0	0	0	0	0	0
21	0	0	0	0	0	0	0	0	0	0	0	0	0	0	0
22	0	0	0	0	0	0	0	0	0	0	0.47	0	0	0	0
23	0	0	0	0		0	0	0	0		0	0	0	0	
24	45.33	0			0	56	0			0	0	0			1.33

**Case**	***KRT6A* F249L**					***KRAS* G12V**				
	**T**	**0 m**	**6 m**	**12 m**	**18 m**	**T**	**0 m**	**6 m**	**12 m**	**18 m**
1	2.53	0		0	0	0	0		0	0
2	0.53			0	0.53	0			0.53	0
3	1.33	0.6				0	0			
4	0	0		0	0	0	0		0	0
5	1.07	0		0.47		0	0		0	
6	0.6	0	0	0.4	0	0	0	0	0	0
7	0	0	0	0		0	0	0	0	
8	0	0			0.53	0	0			0
9	0	0	0	0		0.47	0	0	0	
10	0.47	0			0	0	0			
11	0	0	0	0		0	0	0	0	
12	1.13	1.27				2.2	0			
13	0.67	0.4				0	0			
14	0	0				0	0			
15	0	0	0	0	0	0	0	0	0	0
16	0	0				0	0			
17	0.93	0.6				0	0			0
18	0	0	0	0	1	0	0	0	0	0
19	0.73	0	0.67	0.93	0	0	0	0	0	0

20	0	0	0	0.6	0	24	0	0	0	0
21	0.47	0.6	0.67	0	1.07	0	0	0	0	0
22	0	0.53	0	0		0	0	0	0	
23	0.6	0.67	0	0.53		0	0	0	0	
24	0	0				12	0			

NOTE: T, tumor tissue; 0 m, before treatment; 6 m, at 6 months after treatment; 12 m at 12 months after treatment; 18 m, at18 months after treatment; Data missing, no DNA sample available for the test.

Table S3. Cox regression analysis of survival related to clinicopathological features and gene mutations shown as P value in cervical adenocarcinoma.

**Features and SNV Mutations**	**Intercept 95% CI**	**Slope 95% CI**	**Significance Level**
Age (Grouped)<br>1.≤ 50<br>2. > 50	−0.5506 to 0.52201	−0.2004 to 0.5147	*p* = 0.3719
Stage (Grouped)<br>(FIGO stage)<br>1 = stage IA-IIA<br>2 = stage IIB-IV	−0.8969 to -0.01071	0.1890 to 0.8362	*p* = **0.0034**
Grade (WHO)	−0.3826 to 1.3735	−0.5398 to 0.2695	*p* = 0.4958
Size	−0.4554 to 0.1788	0.03219 to 0.2817	*p* = **0.0160**
LN metastasis	−0.1639 to 1.9972	−0.9884 to 0.1551	*p* = 0.1383
LVSI	0.03166 to 1.4255	−0.7175 to 0.06038	*p* = 0.0926
Recurrence	1.0090 to 1.8481	−0.9516 to -0.4770	*p* < **0.0001**
HPV	−0.6311 to 0.4627	−0.1869 to 0.6711	*p* = 0.2544
p16	−0.3017 to 0.6351	−0.4536 to 0.2870	*p* = 0.6349
PIK3CA - T	−0.1860 to 0.9693	−0.4754 to 0.2376	*p* = 0.4965
PIK3CA - B	0.3483 to 1.5184	−0.7736 to −0.09306	*p* = **0.0150**
PIK3CA - F	0.06986 to 1.1301	−0.6407 to 0.04071	*p* = 0.0806
KRT6A - T	−0.5145 to 0.5844	−0.2376 to 0.4754	*p* = 0.4965
KRT6A - B	−0.6645 to 0.8606	−0.3565 to 0.4938	*p* = 0.7404
KRT6A - F	−0.6697 to 0.6141	−0.2744 to 0.5522	*p* = 0.4848
KRAS - T	−0.5505 to 1.5029	−0.6821 to 0.3964	*p* = 0.5883
KRAS - B	n/a	n/a	n/a
KRAS - F	−2.0705 to 1.6419	−0.7379 to 1.1665	*p* = 0.6349

NOTE: SNV, single nucleotide variation; LVSI, lymphvascular space invasion; -B, Mutation detected in circulating DNA collected before treatment; -F, Mutation detected in circulating DNA collected during follow-up after treatment; -T, Mutation detected in tumor tissue DNA.

Table S4. Cox regression analysis of recurrence related to clinicopathological features and gene mutations shown as *p* value in cervical adenocarcinoma.

**Features and SNV mutations**	**Intercept 95% CI**	**Slope 95% CI**	**Significance level**
Age (Grouped)<br>1. ≤ 50<br>2. > 50	−0.5704 to 0.6275	−0.2136 to 0.5850	*p* = 0.3452
Stage (Grouped)<br>(FIGO stage)<br>1 = stage IA–IIA	−0.7747 to 0.3377	0.01125 to 0.8012	*p* = 0.0561

2 = stage IIB–IV			
Grade (WHO)	−0.7588 to 1.2273	−0.4306 to 0.4847	p = 0.9036
Size	−0.4459 to 0.2681	0.03187 to 0.3128	p = **0.0185**
Pelvic LN metastasis	−0.7555 to 2.2555	−1.0466 to 0.5466	p = 0.5071
LVSI	−0.3152 to 1.4866	−0.6885 to 0.3171	p = 0.4465
HPV	−0.4998 to 0.7524	−0.3543 to 0.6280	p = 0.5693
p16	−0.2983 to 0.9649	−0.6660 to 0.3326	p = 0.4836
PIK3CA - T	0.1383 to 1.3722	−0.6815 to 0.08010	p = 0.1157
PIK3CA - B	0.08342 to 1.5166	−0.7167 to 0.1167	p = 0.1493
PIK3CA - F	0.4216 to 1.5784	−0.8717 to −0.1283	p = **0.0114**
KRT6A - T	−0.5171 to 0.7129	−0.2662 to 0.5319	p = 0.4971
KRT6A - B	−0.8590 to 0.8198	−0.2817 to 0.6542	p = 0.4171
KRT6A - F	−0.6998 to 0.8386	−0.3425 to 0.6481	p = 0.5209
KRAS - T	−0.7753 to 1.5372	−0.6549 to 0.5597	p = 0.8723
KRAS - B	n/a	n/a	n/a
KRAS - F	−2.3293 to 1.7579	-0.7626 to 1.3341	p= 0.5661

NOTE: SNV, single nucleotide variation; LVSI, lymphvascular space invasion; -B, Mutation detected in circulating DNA collected before treatment; -F, Mutation detected in circulating DNA collected during follow-up after treatment; -T, Mutation detected in tumor tissue DNA.

Table S5. Association of survival and with clinicopathological features and gene mutations in cervical adenocarcinoma analyzed using Fisher's exact test shown as p-value.

Features and SNV Mutations	Survival	Recurrence
Age grouped	0.6146	0.3926
Stage grouped	**0.0145**	0.1336
Grade grouped	0.5440	1.0000
Size grouped	0.3394	0.0886
LN metastasis	0.2747	n/a
LVSI	0.1548	0.5696
Recurrence	**0.0005**	n/a
Survival	n/a	0.0005
HPV	0.2705	0.6080
p16	1.0000	1.0000
PIK3CA - T	0.6299	0.1819
PIK3CA - B	**0.0329**	0.1819
PIK3CA - F	0.2105	**0.0325**
KRAS - T	0.5212	1.000
KRAS - B	n/a	n/a
KRAS - F	1.0000	1.0000
KRT6A - T	0.6299	0.6591
KRT6A - B	1.0000	0.6213
KRT6A - F	0.5765	0.6199

Table S6. *PIK3CA* mutation detection in cervical adenocarcinoma with recurrence.

Case	Tumor DNA	cfDNA before treatment	cfDNA at 6th month after treatment	cfDNA at 12th month after treatment	cfDNA at 18th month after treatment	Recurrence time: month after treatment
5	(+)	(−)	n/a	(+)	n/a	13
6	(+)	(+)	(+)	(+)	(+)	36
7	(+)	(+)	(+)	(+)	n/a	29
9	(−)	(+)	(+)	(+)	n/a	10
11	(+)	(−)	(+)	(+)	n/a	23
13	(+)	(+)	n/a	n/a	n/a	13
16	(−)	(−)	n/a	n/a	n/a	25

NOTE: (+), positive; (−), negative; n/a, no sample available for the detection.

Table S7. *PIK3CA* mutation and clinical outcome in cervical adenocarcinoma in previous reports.

Author and Year	Number of case studied	Number of case with *PIK3CA* mutation detected in tumor DNA (%)	Correlation *PIK3CA* mutation with clinical outcome	Methods of *PIK3CA* detection	Purity of tumor cells
**McIntyre JB *et al.* 2013**	13	3 (23.1)	Associated with short OS in Ib/II (not in III/IVa) patients	PCR-Seq	≥75%
**Wright A *et al.* 2013**	40	10 (25.0)	Associated with short OS	Oncomap v4	No information in detail
**Xiang L *et al.* 2014**	106*	11 (10.4%)	Not associated with short PFS	PCR-Seq	≥50%
**Xiang L *et al.* 2015**	101	9 (8.9%)	Associated with longer PFS	PCR-Seq	≥50%
**Akbarov *et al.* 2017**	19	4 (21.1)	No information	Targeted Deep Seq	No information
**Hodgson A *et al.* 2017**	6	53(5.7%)	No information	Targeted Deep Seq	≥50%
**Razia *et al.* 2019**	3	53 (5.7%)	No information	PCR-Seq	≥85%
**Scholl *et al.* 2019**	182	63 (34.6%)	Associated with short PFS, when together with loss of function in epigenetic regulator genes	WES and Targeted Seq	No information

NOTE: * including adenocarcinoma, adenosquamous cell carcinoma and other types of cervical cancer.
